# Coxsackievirus and adenovirus receptor expression in human endometrial adenocarcinoma: possible clinical implications

**DOI:** 10.1186/1477-7819-6-59

**Published:** 2008-06-17

**Authors:** Costas T Giaginis, Apostolos C Zarros, Maria A Papaefthymiou, Aikaterini E Papadopouli, Ioannis K Sfiniadakis, Stamatios E Theocharis

**Affiliations:** 1 Department of Forensic Medicine and Toxicology, Medical School, University of Athens, Greece; 2 Department of Pathology, Naval Hospital, Athens, Greece

## Abstract

The coxsackievirus and adenovirus receptor (CAR) is a crucial receptor for the entry of both coxsackie B viruses and adenoviruses into host cells. CAR expression on tumor cells was reported to be associated with their sensitivity to adenoviral infection, while it was considered as a surrogate marker for monitoring and/or predicting the outcome of adenovirus-mediated gene therapy. The aim of the present study was to evaluate the clinical significance of CAR expression in endometrial adenocarcinoma. CAR expression was assessed immunohistochemically in tumoral samples of 41 endometrial adenocarcinoma patients and was statistically analyzed in relation to various clinicopathological parameters, tumor proliferative capacity and patient survival. CAR positivity was noted in 23 out of 41 (56%) endometrial adenocarcinoma cases, while high CAR expression in 8 out of 23 (35%) positive ones. CAR intensity of immunostaining was classified as mild in 11 (48%), moderate in 10 (43%) and intense in 2 (9%) out of the 23 positive cases. CAR positivity was significantly associated with tumor histological grade (p = 0.036), as well differentiated tumors more frequently demonstrating no CAR expression. CAR staining intensity was significantly associated with tumor histological type (p = 0.016), as tumors possessing squamous elements presented more frequently intense CAR immunostaining. High CAR expression showed a trend to be correlated with increased tumor proliferative capacity (p = 0.057). Patients with tumors presenting moderate or intense CAR staining intensity were characterized by longer survival times than those with mild one; however, this difference did not reach statistical significance. These data reveal, for the first time, the expression of CAR in clinical material obtained from patients with endometrial adenocarcinoma in relation to important clinicopathological parameters for their management. As CAR appears to modulate the proliferation and characteristics of cancer cells, its expression could be considered of possible clinical importance for future (gene) therapy applications.

## Background

The coxsackievirus and adenovirus receptor (CAR) is a 46-kDa transmembrane protein, which functions as a primary receptor for both coxsackie B virus (CVB) and adenovirus (Ad) [1]. This cell surface receptor plays a crucial role in CVB and Ad entry into host cells [2]. CAR mediates homotypic intercellular interactions, while in polarized endothelial cells CAR is closely associated with the tight junction, where it contributes to the barrier of paracellular flow of solutes and macromolecules [3]. A strong correlation of CAR levels with the viral sensitivity of several cell types has been reported [4-6]. In fact, CAR has been shown to be a docking site for Ad, thus acting as a key receptor for the enhancement of the virus-to-host affinity and the initiation of the virus internalization to the host cell [7,8]. On cells lacking CAR, virus uptake takes place with lower efficiency [7,9] due to the existence of a secondary pathway leading to the viral internalization [7,10].

The very promising use of Ad vectors in gene therapy, since Ads are relatively safe, highly infectious, and capable of delivering therapeutic genes to different cell types [10,11], still faces a critical prerequisite, which is no other than the identification of highly efficient and accurate systems for delivering the therapeutic genes into target cells [12]. In this regard, CAR expression could be a surrogate marker for monitoring and/or predicting the outcome of gene therapy, while by increasing CAR levels, resistant cells could become more sensitive to Ad infection [13]. However, only a limited number of studies concerning CAR expression have been made on clinical tissue material. In this aspect, Persson et al. presented an immunohistochemical study in human normal brain and human brain tumors, suggesting that neuroblastomas and medulloblastomas could be suitable for adenovirus-mediated gene therapy [14,15]. Moreover, recent studies have suggested a pathophysiological role for CAR in bladder cancer and glioma cells, rendering CAR as a membrane receptor which conveys its signal into the nucleus and results in cell proliferation suppression [16-18]. These findings raise the question whether CAR expression could be related to the tumor proliferative capacity or differentiation amongst the different tumor cell types.

Endometrial adenocarcinoma is the most common malignant tumor of the female tract and the fourth most common cancer in women following breast, colorectal and lung cancer in the Western world [19]. A substantial decrease in the incidence and mortality of endometrial cancer seems unlikely in the next few years, as early detection and treatment modalities have not been proven to possess a major impact on mortality [20]. Epigenetic modification reagents, including DNA methyltransferase and histone deacetylase inhibitors, when used alone or in combination with conventional chemotherapy, seem to be beneficial for endometrial cancer patients [21]. However, further research advancements are recommended to bring about new strategies and technologies, which ultimately improve the diagnosis and treatment of women with endometrial cancer [21].

In the light of the above considerations, the present study aimed to estimate the immunohistochemical CAR expression in tumoral specimens obtained from endometrial adenocarcinoma patients. We also aimed to evaluate the association of CAR expression and staining intensity with various clinicopathological parameters, tumor proliferative capacity and patient survival.

## Patients and Methods

### Patients

Forty-one endometrial adenocarcinoma specimens obtained from an equal number of patients who underwent surgery due to endometrial cancer were included in this study. None of the patients received any kind of anti-cancer treatment prior to surgery. The mean age of the patient cohort was 63.4 ± 9.6 years (median: 64 years, range: 40–82 years). Tumors were typed according to the presence or not of squamous elements. Three levels of differentiation were used to classify grading as: well, moderately and poorly differentiated. Tumors staging was assessed according to the standards of the Federation Internationationale de Gynecologistes et Obstetricianes (FIGO) [22]. The patients were followed up, with the length of the follow up varying from 22 to 94 months (mean 65.51 ± 16.19 median 64 months). Twenty six patients were followed up until death, while the remaining 15 patients were remained disease free. All the examined clinicopathological parameters are reported in Tables 1, 2 and 3.

**Table 1:** Associations between CAR positivity and clinicopathological characteristics of the 41 patients with endometrial adenocarcinoma

**Clinicopathological characteristics**	**CAR positivity**		
	
	**Negative (%)**	**Positive (%)**	***P*-value**
**Patients**	18 (44)	23 (56)	
**Age **(mean ± SD), years	64.7 ± 7.5	62.4 ± 11.0	0.623
< 63	8 (20)	12 (29)	
≥63	10 (24)	11 (27)	
**pStage (FIGO)**			0.675
I	16 (40)	18 (44)	
II	1 (2)	3 (8)	
III	0 (0)	1 (2)	
IV	1 (2)	1 (2)	
**Histological type**			0.684
Positive for squamous elements	3 (8)	5 (12)	
Negative for squamous elements	15 (36)	18 (44)	
**Histological grade**			**0.036**
Well differentiated	7 (17)	3 (8)	
Moderately differentiated	7 (17)	18 (44)	
Poorly differentiated	4 (10)	2 (4)	
**Ki-67 protein statement**			0.829
Ki-67 below mean (<40%)	10 (24)	12 (29)	
Ki-67 over mean (≥ 40%)	8 (20)	11 (27)	

**Table 2:** Associations between CAR overexpression and clinicopathological characteristics of the 23 CAR positive endometrial adenocarcinoma cases

**Clinicopathological characteristics**	**CAR expression**		
	
	**< 31% (%)**	≥31% (%)	***P*-value**
**Patients**	15 (65)	8 (35)	
**Age **(mean± SD), years	62.9 ± 12.1	61.5 ± 9.6	0.469
< 63	8 (35)	5 (22)	
≥63	7 (30)	3 (13)	
**pStage (FIGO)**			0.147
I	12 (52)	6 (27)	
II	3 (13)	0 (0)	
III	0 (0)	1 (4)	
IV	0 (0)	1 (4)	
**Histological type**			0.181
Positive for squamous elements	2 (9)	3 (13)	
Negative for squamous elements	13 (56)	5 (22)	
**Histological grade**			0.379
Well differentiated	3 (13)	0 (0)	
Moderately differentiated	11 (48)	7 (31)	
Poorly differentiated	1 (4)	1 (4)	
**Ki-67 protein statement**			**0.057**
Ki-67 below mean (<40%)	10 (43)	2 (8)	
Ki-67 over mean (≥ 40%)	5 (22)	6 (27)	

**Table 3:** Associations between CAR staining intensity and clinicopathological characteristics of the 23 CAR positive endometrial adenocarcinoma cases

**Clinicopathological characteristics**	**CAR intensity**			
	
	**Mild (%)**	**Moderate (%)**	**Intense (%)**	***P*-value**
**Patients**	11 (48)	10 (43)	2 (9)	
**Age **(mean ± SD), years	63.2 ± 11.5	63.6 ± 11.2	52.5 ± 0.7	0.359
< 63	6 (26)	5 (22)	2 (9)	
≥63	5 (22)	5 (22)	0 (0)	
**pStage (FIGO)**				0.395
I	7 (30)	9 (39)	2 (9)	
II	3 (14)	0 (0)	0 (0)	
III	0 (0)	1 (4)	0 (0)	
IV	1 (4)	0 (0)	0 (0)	
**Histological type**				**0.016**
Positive for squamous elements	1 (4)	2 (9)	2 (9)	
Negative for squamous elements	10 (44)	8 (34)	0 (0)	
**Histological grade**				0.881
Well differentiated	1 (4)	1 (4)	1 (4)	
Moderately differentiated	9 (40)	7 (30)	1 (5)	
Poorly differentiated	1 (4)	2 (9)	0 (0)	
**Ki-67 protein statement**				0.555
Ki-67 below mean (< 40%)	7 (31)	4 (17)	1 (4)	
Ki-67 over mean (≥ 40%)	4 (17)	6 (26)	1 (5)	

### Immunohistochemistry

Immunostainings for CAR was performed on paraffin-embedded tissue sections using a commercially available rabbit *anti*-CAR monoclonal antibody (CAR H300, Santa Cruz Biochemicals, Santa Cruz, CA, USA). Briefly, 4 μm thick tissue sections were dewaxed in xylene and were brought to water through graded alcohols. To remove the endogenous peroxidase activity, sections were then treated with freshly prepared 0.3% hydrogen peroxide in methanol in the dark, for 30 minutes (min), at room temperature. Non-specific antibody binding was then blocked using Snipper, a specific blocking reagent for mouse primary antibodies (Sniper, Biocare Medical, Walnut, Creek, CA, USA) for 5 min. The sections were then incubated for 1 hour (h), at room temperature, with the primary antibody, CAR, diluted 1:100, respectively, in phosphate buffered saline (PBS). After washing three times with PBS, sections were incubated at room temperature with biotinylated linking reagent (Biocare Medical) for 10 min, followed by incubation with peroxidase-conjugated streptavidin label (Biocare Medical) for 10 min. The resultant immune peroxidase activity was developed in 0.5% 3,3'-diaminobenzidine hydrochloride (DAB; Sigma, Saint Louis, MO, USA) in PBS containing 0.03% hydrogen peroxide for 3 min. Sections were counterstained with Harris' hematoxylin and mounted in Entellan (Merck, Darmstadt, Germany). An additional step of antigen retrieval (citrate buffer at pH 6.1 and microwave heating) was performed before incubation with the primary *anti*-CAR antibody. Appropriate negative controls were performed by omitting the primary antibody and/or substituting it with an irrelevant anti-serum. As positive control, colon cancer tissue sections with known increased CAR positivity were used [23]. The tumor proliferative capacity was assessed immunohistochemically, using a mouse *anti*-human Ki-67 antigen; IgG_1k _antibody (clone MIB-1, Dakopatts) as previously described [24].

### Evaluation of immunohistochemistry

The percentages of positively stained cells were obtained by counting at least 1000 tumor cells in each case by two independent observers (SET and IKS) blinded to the clinical data with complete observer agreement. Specimens were considered "positive" for CAR and Ki-67 when more than 5% of the tumor cells were stained, while they were characterized to present "high" CAR and Ki-67 expression when the percentage of positively stained cells exceeded the mean percentage value. The intensity of CAR immunostaining was also estimated and graded on a three step scale as: mild (+), moderate (++) and intense (+++). The cellular pattern of distribution of CAR immunostaining was characterized as membraneous and cytoplasmic. All endometrial adenocarcinoma cases were Ki-67 positive, presenting nuclear pattern of staining.

### Statistical analysis

Chi-square tests were used to assess the association of CAR positivity, overexpression and staining intensity with clinicopathological variables and tumor proliferative capacity. Survival curves were constructed using the Kaplan-Meier method and compared using the log-rank test. Cox proportional hazard regression analysis was used to evaluate the effect of CAR positivity, level of expression (low vs high level of CAR expression) and staining intensity as prognostic factors on patient survival. A 2-tailed P < 0.05 was considered (statistically) significant. Statistical analyses were performed using the software package SPSS for Windows (version 11.0; SPSS Inc., Chicago, IL, USA).

## Results

**Figure 1:**
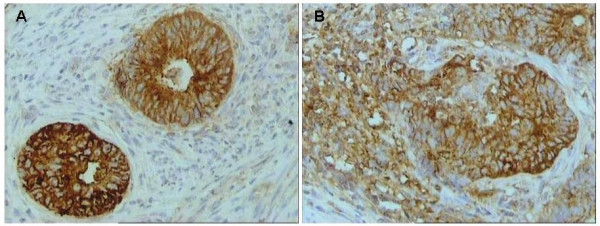
Intense immunostaining for CAR in tumor cells in representative endometrial adenocarcinoma cases (original magnification ×200). A. Negative for squamous elements. B. Positive for squamous elements.

CAR positivity was noted in 23 out of 41 (56%) of the examined endometrial adenocarcinoma cases (Table 1). Representative CAR immunostaining is presented in Figure 1. The pattern of CAR distribution was both cytoplasmic and membraneous in all positive cases examined. High CAR expression was noted in 8 out of 23 (35%) of the positive cases (Table 2). The intensity of CAR immunostaining was classified as mild in 11 (48%), moderate in 10 (43%) and intense in 2 (9%) out of 23 positive cases (Table 3).

CAR positivity was significantly associated with tumor histological grade, as well differentiated tumors most frequently presenting no CAR expression compared to moderately and poorly differentiated ones. (*P *= 0.036, Table 1). High CAR expression showed a trend to be correlated with increased proliferative capacity (*P *= 0.057, Table 2). CAR staining intensity was significantly associated with tumor histological type, as cases possessing squamous elements presented more frequently intense CAR immunostaining (*P *= 0.016, Table 3). CAR positivity, level of expression and staining intensity were not significantly associated with the other clinicopathological parameters examined (Tables 1, 2 and 3).

The Kaplan-Meier product-limit method for overall analysis survival according to CAR positivity (positive *vs *negative CAR staining), level of expression (high *vs *low level of CAR expression) and staining intensity (mild *vs *moderate and intense CAR staining intensity) in patients with endometrial adenocarcinoma did not reveal statistically significant correlations (log-rank test, *P *= 0.799, *P *= 0.816 and *P *= 0.127, respectively) (data not shown). The survival of patients with tumors presenting moderate or intense CAR staining intensity (mean survival rate 72.3 ± 11.0 months) was longer than those presenting mild intensity (mean survival rate 55.6 ± 19.5 months); however the difference did not reach statistical significance in univariate analysis (*P *= 0.127, data not shown).

## Discussion

The expression of CAR in human tumors and tumor cell lines has been subject of several studies [5,6,25-28] which have detected this transmembrane protein in variable and often low levels. The present study focused on the immunohistochemical examination of CAR expression in endometrial adenocarcinoma samples and revealed the clinical significance of CAR in certain aspects of endometrial neoplasia, such as tumor differentiation, histological type and proliferation. More to the point, CAR positivity was noted in 56% of the examined cases. This incidence of endometrial adenocarcinoma CAR positivity cannot be considered among the highest ever found on tumor malignancies, since Gu et al. have observed a 75% CAR positivity on osteosarcoma samples; however, it is certainly higher than that of lung adenocarcinoma [29,30]. To this point, it should be noted the effectiveness of adenoviral gene therapy depends on the amount of CAR expression on target cells. Thus, the current study reinforced the suitability for adenoviral gene therapy in the case of endometrial adenocarcinoma.

Our study is the first report examining the clinical significance of CAR expression in patients with endometrial adenocarcinoma. We found that well differentiated tumors more frequently presenting no CAR expression compared to moderately and poorly differentiated ones. In this aspect, Korn et al. also revealed a significant association between CAR expression and tumor histological grade in patients with gastrointestinal malignancies; however, moderately to poorly differentiated tumors more frequently demonstrating low or no CAR expression [31]. CAR expression was also reported to be increased with increasing grade of tumor in breast cancer patients; however, this difference was not statistically significant [32]. Moreover, in the current study, high CAR expression showed a trend to be correlated with increased proliferative capacity. In this context, CAR expression was reported to modulate the proliferative capacity of cancer cells, *in vitro *[13,16,18,33]. In fact, the presence of CAR was found not only to facilitate viral uptake of adenovirus, but also to inhibit cell growth in bladder cancer and malignant glioma cells [13,16,18,33]. The latter is not in line with the current findings and could be ascribed to the individual characteristics among the different types of cancer. Moreover, our results were based on the *in situ *detection of CAR protein by immunohistochemistry, while the potential tumor suppressor role of CAR was reported for cultured cell lines or tumor cells injected into nude mice [13,16,18,33]. We also found that CAR staining intensity was significantly associated with the tumor histological type. This result is in line with previous evidence where higher rates of CAR expression were detected in lung squamous cell carcinoma than in adenocarcinoma [30].

To our knowledge, there is limited data so far highlighting to the prognostic value of CAR expression in cancer. In this respect, our study is the first report examining the clinical significance of CAR expression in the prognosis of patients with endometrial adenocarcinoma. We did not found any significant association of CAR positivity, level of expression and staining intensity with patient survival. It should be noted that the survival of patients with tumors presenting moderate or intense CAR staining (mean survival rate 72.3 ± 11.0 months) was longer than those with mild (mean survival rate 55.6 ± 19.5 months); however, this difference did not reach statistically significance in univariate analysis (*P *= 0.127). In this context, Martin et al. showed that elevated levels of CAR expression were significantly associated with poor overall survival in patients with breast cancer [32]. It has also been shown that the soluble splice variants CAR 3/7 and CAR 4/7, but no the full-length hCAR were of independent prognostic relevance for progression-free or overall survival of ovarian cancer patients [34].

In the last few years, the use of adenovirus vectors is gaining increasingly interesting in order to advance new therapeutic approaches against cancer. Thus, many tumor samples have been examined for CAR expression, which has generally been found to correlate with susceptibility to transduction [5,18,28,35]. In several human malignancies, including bladder and prostate carcinoma and glioblastoma, CAR expression was downregulated during the progression to malignancy [16,36,37]. In CAR-deficient prostate and glioma tumor cell lines, expression of CAR by transfection resulted in suppression of cell proliferation and decreased tumorogenicity [18,33]. CAR expression also inhibited cell proliferation and was associated with modulations in the activity of the cell cycle regulators p21-PIC and Rb in bladder cancer cells [16]. Importantly, CAR dependent growth inhibition required the presence of CAR-specific antibody which blocked homotypic adhesion [16].

A strong correlation of CAR levels with the viral sensitivity of any given cell has been reported [4-6]. Although the normal cellular function of CAR is not known, some researchers have suggested that CAR may serve as a cell-cell adhesion molecule [38], while others have shown an *in vitro *and *in vivo *tumor-suppressive role for CAR [33]. It is thought that CAR can inhibit cancer growth by behaving as a membrane receptor, which conveys its signal into the nucleus, thus resulting in suppression of the proliferative mechanisms [13]. Moreover, reduced CAR expression was shown to induce lung metastasis [39]. Overall, correlating the CAR expression in all known tumor malignancies with clinicopathological parameters cannot only provide crucial information about its role in malignant transformation, but it can also establish a better view for future gene therapy approaches. Currently, Othman et al. reported that endometriosis cells expressed higher levels of CAR mRNA as compared with normal endometrial cells [40]. In addition, it was shown that adenoviruses can effectively transfect endometriosis cells *in vitro*. The dominant negative mutants of Estrogen receptors (DN-ER) delivered to endometriosis cells via an adenovirus decreased cell proliferation, induced apoptosis and suppressed cytokine production by these cells [40]. Such data supported substantial evidence that adenovirus-mediated delivery of DN-ER to endometriosis cells can be a potential therapeutic approach for endometriosis [40].

## Conclusion

The data presented in this study revealed enhanced CAR expression in endometrial adenocarcinoma specimens. CAR protein expression was associated with important clinicopathological parameters with respect to the diagnosis of patients with endometrial cancer. Although CAR protein failed to predict patient survival, the current study supports evidence for potential implication of CAR protein in endometrial carcinogenesis. The use of Ad vectors in gene therapy needs an efficient and accurate system for delivering the therapeutic gene into target cells [12]. In this regard, CAR expression could be a surrogate marker for monitoring and/or predicting the outcome of gene therapy, while its increase might contribute to the upregulation of cellular sensitivity towards Ad infection [13]. It is, however, without doubt that in order to understand the physiological role of CAR in cellular function and proliferation, a systematic approach towards the identification of its natural ligand(s) should also be attempted.

## Competing interests

The authors declare that they have no competing interests.

## Authors' contributions

CTG participated in the design of the study, drafted the paper and performed the statistical analysis, ACZ participated in the statistical analysis and drafted the paper, MAP contributed to the immunostainings and clinical data collection, AEP contributed to the immunostainings and clinical data collection, IKS carried out the immunohistochemistry data evaluation, SET designed the study, carried out the immunohistochemistry data evaluation and corrected the manuscript. All authors read and approved the final manuscript.

## References

[B1] Bowles KR, Gibson J, Wu J, Shaffer LG, Towbin JA, Bowles NE (1999). Genomic organization and chromosomal localization of the human coxsackievirus B-adenovirus receptor gene. Hum Genet.

[B2] Bergelson JM, Cunningham JA, Droguett G, Kurt-Jones EA, Krithivas A, Hong JS, Horwitz MS, Crowell RL, Finberg RW (1997). Isolation of a common receptor for Coxsackie B viruses and adenoviruses 2 and 5. Science.

[B3] Coyne CB, Bergelson JM (2005). CAR: A virus receptor within the tight junction. Adv Drug Deliv Rev.

[B4] Kim JS, Lee SH, Cho YS, Choi JJ, Kim YH, Lee JH (2002). Enhancement of the adenoviral sensitivity on human ovarian cancer cells by transient expression of coxsackievirus and adenovirus receptor (CAR). Gynecol Oncol.

[B5] Hemmi S, Geertsen R, Mezzacasa A, Peter I, Dummer R (1998). The presence of human coxsackievirus and adenovirus receptor is associated with efficient adenovirus-mediated transgene expression in human melanoma cell cultures. Hum Gene Ther.

[B6] Li Y, Pong RC, Bergelson JM, Hall MC, Sagalowsky AI, Tseng CP, Wang Z, Hsieh JT (1999). Loss of adenoviral receptor expression in human bladder cancer cells: a potential impact on the efficacy of gene therapy. Cancer Res.

[B7] Leon RP, Hedlund T, Meech SJ, Li S, Schaack J, Hunger SP, Duke RC, DeGregori J (1998). Adenoviral-mediated gene transfer in lymphocytes. Proc Natl Acad Sci USA.

[B8] Wang X, Bergelson JM (1999). Coxsackievirus and adenovirus receptor cytoplasmic and transmembrane domains are not essential for coxsackievirus and adenovirus infection. J Virol.

[B9] Bai M, Campisi L, Freimuth P (1994). Vitronectin receptor antibodies inhibit infection of HeLa and A549 cells by adenovirus type 12 but not by adenovirus type 2. J Virol.

[B10] Barnett BG, Crews CJ, Douglas JT (2002). Targeted adenoviral vectors. Biochim Biophys Acta.

[B11] Zhang WW (1999). Development and application of adenoviral vectors for gene therapy of cancer. Cancer Gene Ther.

[B12] Hedley SJ, Chen J, Mountz JD, Li J, Curiel DT, Korokhov N, Kovesdi I (2006). Targeted and shielded adenovectors for cancer therapy. Cancer Immunol Immunother.

[B13] Okegawa T, Li Y, Pong RC, Hsieh JT (2002). Cell adhesion proteins as tumor suppressors. J Urol.

[B14] Persson A, Fan X, Widegren B, Englund E (2006). Cell type- and region-dependent coxsackie adenovirus receptor expression in the central nervous system. J Neurooncol.

[B15] Persson A, Fan X, Salford LG, Widegren B, Englund E (2007). Neuroblastomas and medulloblastomas exhibit more coxsackie adenovirus receptor expression than gliomas and other brain tumors. Neuropathology.

[B16] Okegawa T, Pong RC, Li Y, Bergelson JM, Sagalowsky AI, Hsieh JT (2001). The mechanism of the growth-inhibitory effect of coxsackie and adenovirus receptor (CAR) on human bladder cancer: a functional analysis of car protein structure. Cancer Res.

[B17] Huang KC, Altinoz M, Wosik K, Larochelle N, Koty Z, Zhu L, Holland PC, Nalbantoglu J (2005). Impact of the coxsackie and adenovirus receptor (CAR) on glioma cell growth and invasion: requirement for the C-terminal domain. Int J Cancer.

[B18] Kim M, Sumerel LA, Belousova N, Lyons GR, Carey DE, Krasnykh V, Douglas JT (2003). The coxsackievirus and adenovirus receptor acts as a tumour suppressor in malignant glioma cells. Br J Cancer.

[B19] Prat J, Gallardo A, Cuatrecasas M, Catasus L (2007). Endometrial carcinoma: pathology and genetics. Pathology.

[B20] Amant F, Moerman P, Neven P, Timmerman D, Limbergen EV, Vergote I (2007). Treatment modalities in endometrial cancer. Curr Opin Oncol.

[B21] Zhou XC, Dowdy SC, Podratz KC, Jiang S-W (2007). Epigenetics considerations for endometrial cancer prevention, diagnosis and treatment. Gynecol Oncol.

[B22] International Federation of Gynecology and Obstetrics (1989). FIGO stages: 1988 revision. Gynecol Oncol.

[B23] Theocharis S, Papaefthymiou M, Giaginis C, Gatzidou E, Vgenopoulou S, Sfiniadakis I, Kouraklis G (2007). CAR expression in gastrointestinal and pancreatic adenocarcinoma. Virchows Archiv.

[B24] Giaginis C, Davides D, Zarros A, Noussia O, Zizi-Serbetzoglou A, Kouraklis G, Theocharis S (2008). Clinical significance of tumor-associated antigen RCAS1 expression in human pancreatic ductal adenocarcinoma. Dig Dis Sci.

[B25] Cripe TP, Dunphy EJ, Holub AD, Saini A, Vasi NH, Mahller YY, Collins MH, Snyder JD, Krasnykh V, Curiel DT, Wickham TJ, DeGregori J, Bergelson JM, Currier MA (2001). Fiber knob modifications overcome low, heterogeneous expression of the coxsackievirus-adenovirus receptor that limits adenovirus gene transfer and oncolysis for human rhabdomyosarcoma cells. Cancer Res.

[B26] Dmitriev I, Krasnykh V, Miller CR, Wang M, Kashentseva E, Mikheeva G, Belousova N, Curiel DT (1998). An adenovirus vector with genetically modified fibers demonstrates expanded tropism via utilization of a coxsackievirus and adenovirus receptor-independent cell entry mechanism. J Virol.

[B27] Fechner H, Wang X, Wang H, Jansen A, Pauschinger M, Scherubl H, Bergelson JM, Schultheiss HP, Poller W (2000). Trans-complementation of vector replication versus Coxsackie-adenovirus-receptor overexpression to improve transgene expression in poorly permissive cancer cells. Gene Ther.

[B28] Miller CR, Buchsbaum DJ, Reynolds PN, Douglas JT, Gillespie GY, Mayo MS, Raben D, Curiel DT (1998). Differential susceptibility of primary and established human glioma cells to adenovirus infection: targeting via the epidermal growth factor receptor achieves fiber receptor-independent gene transfer. Cancer Res.

[B29] Gu W, Ogose A, Kawashima H, Ito M, Ito T, Matsuba A, Kitahara H, Hotta T, Tokunaga K, Hatano H, Morita T, Urakawa S, Yoshizawa T, Kawashima H, Kuwano R, Endo N (2004). High-level expression of the coxsackievirus and adenovirus receptor messenger RNA in osteosarcoma, Ewing's sarcoma and benign neurogenic tumors among musculoskeletal tumors. Clin Cancer Res.

[B30] Wang Y, Wang S, Bao Y, Ni C, Guan N, Zhao J, Salford LG, Widegren B, Fan X (2006). Coxsackievirus and adenovirus receptor expression in non-malignant lung tissues and clinical cancers. J Mol Histol.

[B31] Korn WM, Christian MM, Lacher MD, McMillian A, Rauen KA, Warren RS, Ferrell L (2006). Expression of the coxsackievirus- and adenovirus receptor in gastrointestinal cancer correlates with tumor differentiation. Cancer Gene Ther.

[B32] Martin TA, Watkins G, Jiang WG (2005). The coxsackievirus adenovirus receptor has elevated expression in human breast cancer. Clin Exp Med.

[B33] Okegawa T, Li Y, Pong RC, Bergelson JM, Zhou J, Hsieh JT (2000). The dual impact of coxsackie and adenovirus receptor expression on human prostate cancer gene therapy. Cancer Res.

[B34] Reimer D, Steppan I, Wiedemair A, Concin N, Hofstetter G, Marth C, Müller-Holzner E, Zeimet AG (2007). Soluble isoforms but not the transmembrane form of coxsackie-adenovirus receptor are of clinical relevance in epithelial ovarian cancer. Int J Cancer.

[B35] Qin M, Chen S, Yu T, Escuadro B, Sharma S, Batra RK (2003). Coxsackievirus adenovirus receptor expression predicts the efficiency of adenoviral gene transfer into non-small cell lung cancer xenografts. Clin Cancer Res.

[B36] Sachs MD, Rauen KA, Ramamurthy M, Dodson JL, De Marzo AM, Putzi MJ, Schoenberg MP, Rodriguez R (2002). Integrin alpha(v) and coxsackie adenovirus receptor expression in clinical bladder cancer. Urology.

[B37] Fuxe J, Liu L, Malin S, Philipson L, Collins VP, Pettersson RF (2003). Expression of the coxsackie and adenovirus receptor in human astrocytic tumors and xenografts. Int J Cancer.

[B38] Honda T, Saitoh H, Masuko M, Katagiri-Abe T, Tominaga K, Kozakai I, Kobayashi K, Kumanishi T, Watanabe YG, Odani S, Kuwano R (2000). The coxsackievirus-adenovirus receptor protein as a cell adhesion molecule in the developing mouse brain. Brain Res Mol Brain Res.

[B39] Yamashita M, Ino A, Kawabata K, Sakurai F, Mizuguchi H (2007). Expression of coxsackie and adenovirus receptor reduces the lung metastatic potential of murine tumor cells. Int J Cancer.

[B40] Othman E-ER, Salama S, Ismail A, Al-Hendy A (2007). Toward gene therapy of endometriosis: adenovirus-mediated delivery of dominant negative estrogen receptor genes inhibits cell proliferation, reduces cytokine production, and induces apoptosis of endometriotic cells. Fertil Steril.

